# How integration of dementia services is improving timely dementia diagnosis: an example from Kent. The author wants to share a programme of work from Kent U.K , where system-wide transformation of services has enabled improved time to diagnosis

**DOI:** 10.1192/j.eurpsy.2023.414

**Published:** 2023-07-19

**Authors:** A. Qazi

**Affiliations:** Kent and Medway NHS and Social care partnership trust, Maidstone, United Kingdom

## Abstract

**Introduction:**

The author wants to share a programme of work from Kent, a County in the south-east of England, where integration of services has enabled system wide quality improvement and innovation to achieve improved time to diagnosis, by best use of available resources and increased diagnostic capacity by engaging with primary care.

**Objectives:**

Improving dementia diagnosis rate and improved time to diagnosis for people seeking help for memory problems.

**Methods:**

The main elements of improving dementia diagnosis rate and improved time to diagnosis are:

1) Pre-diagnostic support through appointment of Dementia Coordinators

2) Transformation of memory services using Quality Improvement methodology to an Enhanced memory assessment and intervention model, which includes diagnosis within six weeks of the GP referral by offering assessment and diagnosis on the same day by clinicians as opposed to a lead time of 18 weeks previously.

3) Increased dementia diagnosis capacity by training primary care colleagues and creating GP with extended roles posts to diagnose non-complex dementia referrals.

4) Introducing a screening tool to diagnose people with dementia in care homes avoiding the need to refer to secondary care services.

5) Having a shared electronic patient record system across the county which enables quick and easy access to patient records.

**Results:**

In the UK dementia diagnosis rates, dropped from 67.6% in February 2020 to 63.2% in December 2020. Post COVID-19 recovery in dementia diagnosis is happening across the country and Kent is ahead at 9% with increase in dementia diagnosis rates compared to a national average of 0.5% increase.

**Conclusions:**

The integrated care system in Kent has enabled collaborative working across organisations to improved dementia diagnosis rates at a fast pace, an example for other health care systems.

In Kent a county-wide, Dementia Special interest group has provided the platform to introduce innovation collaboratively across the entire county and has made a significant difference to people with dementia across the whole pathway of care that has particularly improved time to diagnosis.

**Disclosure of Interest:**

None Declared

